# Evidence from Thermal Aging Indicating That the Synergistic Effect of Glyoxal and Sodium Sulfite Improved the Thermal Stability of Conformational Modified Xanthan Gum

**DOI:** 10.3390/polym14020243

**Published:** 2022-01-07

**Authors:** Shuai Yuan, Jiayuan Liang, Yanmin Zhang, Hongyu Han, Tianyi Jiang, Yang Liu, Yonggang Zhang, Wei Wang, Xueqian Dong

**Affiliations:** 1 School of Bioengineering, Qilu University of Technology (Shandong Academy of Sciences), Jinan 250353, China; 17854117125@163.com; 2 School of Food Science and Engineering, Qilu University of Technology (Shandong Academy of Sciences), Jinan 250353, China; jiayuanl1004@163.com (J.L.); zhangyanmin201314@163.com (Y.Z.); hanhongyudlut@gmail.com (H.H.); liuyang0219@hotmail.com (Y.L.); zyg8500@126.com (Y.Z.); wangwei87@qlu.edu.cn (W.W.); 3 Shandong Food Ferment Industry & Design Institute, Jinan 250013, China; 4 School of Municipal and Environmental Engineering, Shandong Jianzhu University, Jinan 250101, China; jiangtianyi12@163.com

**Keywords:** xanthan gum, thermal stability, genetically engineering, pyruvyl, glyoxal

## Abstract

Xanthan gum is prone to thermal oxidative degradation, which limits its applications. However, conformational changes in xanthan gum and appropriate stabilizers may improve its thermal stability. Therefore, in this study, we aimed to establish a strategy to maintain the viscosity of xanthan gum during long-term storage at high temperatures. We modified the original strain used for xanthan gum production by genetic engineering and added stabilizers during the production process. The structure and thermal stability of the resulting xanthan gum samples were then determined. Pyruvyl deficiency, combined with the addition of sodium sulfite and glyoxal during the production process, was found to significantly improve the maintenance of viscosity. The apparent viscosity of the new xanthan gum solution remained above 100 mPa·s after being stored at 90 °C for 48 days. Fourier-transform infrared spectra and scanning electron microscopy images showed that pyruvate-free xanthan gum with added stabilizers had more extensive cross-linking than natural xanthan gum. In conclusion, these findings may contribute to the use of xanthan gum in applications that require high temperatures for a long period of time.

## 1. Introduction

Xanthan gum is an acidic polysaccharide produced by *Xanthomonas campestris*. The main chain of xanthan gum comprises two d-glucose molecules connected by a β-1,4-glucosidic bond. Every second glucose residue on the main chain is connected to a trisaccharide side chain composed of an internal d-mannose, a d-glucuronic acid, and an external d-mannose [1,2]. Most of the internal mannose residues of natural xanthan gum are acetylated, and the external mannose residues are either acetylated or pyruvylated [2]. An aqueous solution of xanthan gum has a variety of unique properties, such as a high salt concentration tolerance, high viscosity (at low concentrations), and good stability under high mechanical shear stress [3,4]. Due to these properties, xanthan gum has many applications, such as in the petroleum, food, and cosmetics industries [5,6]. Xanthan gum is one of the essential biopolymers used in the petroleum industry [7]. However, after its use in oil displacement for several months, xanthan gum is easily oxidized and degraded at high temperatures, which limits its applications [7,8]. Therefore, the development of technologies to maintain the viscosity of xanthan gum at high temperatures is important to the petroleum industry.

Many studies have aimed to improve the thermal stability of xanthan gum. Its thermal stability is enhanced by hydrophobic modification [9], cross-linking [10], grafting [11], and maintaining a neutral pH in solution [12]. However, adding deoxidizers, such as formate [13] or Na_2_SO_3_ [14], is currently the most common method used to improve the thermal stability of the polymer. Moreover, the effects of deoxidizers may be further enhanced by the addition of other reagents, such as glyoxal. Plank et al. (2007) [15] added glyoxal and Na_2_SO_3_ simultaneously to several biopolymers and found that the thermal stability of many was enhanced, but the thermal stability of xanthan gum did not change significantly. The underlying mechanism for this difference in thermal stability has not been determined, but it is thought to be related to the unique internal structure of xanthan gum [8,16].

In addition to the aforementioned biochemical methods, several studies have found that the ordered conformation of xanthan gum is responsible for its thermal stability [17]. The acetyl and pyruvyl groups on the side chains of xanthan gum significantly affect its conformation. Specifically, pyruvyl groups disrupt the ordered conformation, whereas acetyl groups stabilize it [18,19]. This pattern provides a new strategy to improve the thermal stability of xanthan gum by changing the acetyl and pyruvyl contents. The *X. campestris* genes responsible for side-chain modification have been extensively studied. The gene *gumF* encodes acetyltransferase I, which is responsible for internal mannose acetylation; *gumG* encodes acetyltransferase II; and *gumL* encodes pyruvate transferase, which is responsible for external mannose modification [20,21]. This information allows us to improve *X. campestris* strains through genetic engineering to achieve the mass production of side chain-modified xanthan gum. The addition of a stabilizer to xanthan gum produced by a genetically engineered strain is likely to further improve its thermal stability.

Therefore, in this study, we used *X. campestris* as the original strain and constructed a pyruvate-deficient mutant (XC-Δ*gumL*) by disrupting the pyruvate transferase-encoding gene *gumL* to achieve the mass production of pyruvate-free xanthan gum. The thermal stabilities of natural xanthan gum (XG), pyruvate-free xanthan gum (XG-L), natural xanthan gum with a deoxidizer and glyoxal added (XG-HC), and pyruvate-free xanthan gum with a deoxidizer and glyoxal added (XG-LC) were measured. Finally, Fourier-transform infrared (FTIR) spectroscopy and scanning electron microscopy (SEM) were used to characterize the structures of XG, XG-L, XG-HC, and XG-LC, with the aims of developing thermostable xanthan gum products and laying a foundation for future investigations of the underlying mechanisms of xanthan gum thermostability.

## 2. Materials and Methods

### 2.1. Bacterial Strains and Culture

*X. campestris* NRRL B-1459 (strain XC) was provided by the Shandong Food Fermentation Industry Research and Design Institute (Jinan, China). The basic media used to culture strain XC and the mutant strains included HYG (HY medium with 10.0 g/L glucose; HY: 5.0 g/L peptone, 3.0 g/L beef powder, 0.5 g/L yeast extract, and 2.0 g/L NaCl, pH 7.0), HYS (HY medium with 150.0 g/L sucrose), and seed culture medium (20.0 g/L sucrose, 5.0 g/L beef powder, 3.0 g/L peptone, and 1 g/L yeast extract; pH 7.0; [22]. Moreover, a starch medium (40.0 g/L corn starch, 6.0 g/L peptone, and 2.0 g/L CaCO_3_) was used for the fermentation-based production of xanthan gum. Strain XC and the mutant strains were cultured at 28 °C.

*Escherichia coli* strains were cultured at 37 °C in Luria–Bertani medium. When required, antibiotics were added (kanamycin, 50 mg/mL).

### 2.2. Construction of X. Campestris Mutants

The *gumL* gene was inactivated by double-crossover homologous recombination with the suicide vector pK18*mobsacB* (kan^R^) [23]. Using genomic DNA extracted from *X. campestris* NRRL B-1459 as a template, regions 260 bp upstream and 229 bp downstream of the *gumL* gene were amplified as homologous arms using *gumL*-F1/*gumL*-R1 and *gumL*-F2/*gumL*-R2 primer pairs (Table 1). The two homologous arms were connected by overlap extension PCR using the primers *gumL*-F1 and *gumL*-R2 to form Δ*gumL*. Δ*gumL* was cloned into pK18mobsacB (kan^R^) using the BamH1 and HindIII restriction enzyme sites to form the plasmid pK18*mobsacB* (kan^R^)-Δ*gumL.* This plasmid was then introduced into *X. campestris* NRRL B-1459 using the electroshock transformation method described by Wang et al. (2016) [24]. Single-exchange mutants were selected on an HYG solid medium containing 50 μg/mL kanamycin. The pyruvate transferase-deletion mutant XC-Δ*gumL* was identified in the HYS medium by PCR using the primers *gumL*-F1 and *gumL*-R1. All plasmid constructs and PCR products were verified by sequencing using corresponding primers.

### 2.3. Preparation of Xanthan Gum Samples

Strain XC and its mutant, XC-Δ*gumL*, were inoculated into 100 mL of seed culture medium in 500 mL shake flasks and cultured at 28 °C and 200 rpm for 24 h. The seed cultures were then inoculated into 80 mL of starch medium in 500 mL shake flasks with 10% (*v*/*v*) bacterial suspension and cultured at 28 °C and 300 rpm for 72 h. The resulting fermentation broth was diluted 10-fold and centrifuged at 10,000× *g* for 30 min to remove cells. After centrifugation, 95% ethanol was added to the supernatant and the precipitate was collected and washed again with 95% ethanol to precipitate the xanthan gum. Finally, the xanthan gum was dried at 60 °C for 6 h.

### 2.4. Determination of Acetyl and Pyruvyl Contents

The acetyl and pyruvyl contents of different xanthan gum samples were determined according to the method described by Cheetham and Punruckvong (1985) [25], with some modifications.

A xanthan gum solution was prepared at a concentration of 0.5% (*m*/*v*) in distilled water. One milliliter of the xanthan gum solution was added to 1 mL of potassium hydroxide solution (0.2 M), and the tube was filled with nitrogen, sealed, and incubated at 45 °C for 6 h. A 5 mL solution of phosphoric acid (0.1 M, 1 mL) and distilled water was accurately prepared and filtered through a 0.22 μm membrane to obtain the acetyl determination solution. 

In total, 1 mL of 0.1 M phosphoric acid was added to the 0.5% polysaccharide solution (1 mL), and the samples were sealed and held at 90 °C for 90 min. Deionized water was added to the sample to a final volume of 5 mL, and the sample was filtered through a 0.22 μm membrane to obtain the pyruvate determination sample. 

Standard curves were plotted with different concentrations of pyruvate (0.005, 0.01, 0.02, 0.04, and 0.08 mg/mL) and acetic acid (0.05, 0.1, 0.2, 0.4, and 0.8 mg/mL).

The acetyl and pyruvyl contents of different xanthan gum samples were measured by high-performance liquid chromatography (HPLC, Agilent Technologies Co., Ltd., Santa Clara, CA, USA). The details were as follows: an Aminex HPX87H column (Bio-Rad, Hercules, CA, USA), 5 mM sulfuric acid as the mobile phase, a flow rate of 0.6 mL/min, a column temperature of 60 °C, and detection at 210 nm. 

The acetyl and pyruvyl contents are calculated according to the following formulae.

$$\mathrm{Pyruvyl}\;\mathrm{content}\;\left(\mathrm{wt}.\%\right)=\frac{Y\times a}{b}\times 100\%\;\mathrm{Acetyl}\;\mathrm{content}\;\left(\mathrm{wt}.\%\right)=\frac{Y\times a}{b}\times \frac{M\left(\mathrm{acetyl}\right)}{M\left(\mathrm{acetic}\;\mathrm{acid}\right)}\times 100\%$$

Here *Y* represents the concentration of acetyl or pyruvyl (g/L) obtained by HPLC analyses, ‘*a*’ was the dilution ratio, and ‘*b*’ was the concentration of determination solution (g/L).

### 2.5. Determination of the Molecular Weight of Xanthan Gum Samples

The molecular weights of different xanthan gum samples were measured according to the method described by Wang et al. (2017) [26], with some modifications. In brief, xanthan gum solutions were prepared at a concentration of 0.1% (*m*/*v*) in distilled water and centrifuged at 10,000× *g* for 30 min. The supernatants were filtered through a 0.45 μm membrane and then a 0.22 μm membrane to prepare the sample. Molecular weights were determined using an Agilent 1260 gel exclusion chromatography (GPC) system (Agilent Technologies, Inc., Santa Clara, CA, USA) with a differential refraction detector and a multi-angle laser scattering detector. NaNO_3_ (0.1%) was used as the eluent at a flow rate of 1 mL/min at 30 °C. Agilent GPC/SEC software was used for data acquisition and analysis.

### 2.6. Determination of the Thermal Stability of Xanthan Gum Samples

The thermal stability of different xanthan gum samples was measured according to the method described by Lambert and Rinaudo (1985) [8], with some modifications. Briefly, XG and XG-L solutions were prepared at a concentration of 0.3% (*m*/*v*) in distilled water and stirred at 1200 rpm until completely dissolved. Na_2_SO_3_ and glyoxal were added to the XG and XG-L solutions to final concentrations of 0.3% and 0.1%, respectively, to prepare XG-HC and XG-LC solutions [15]. The prepared samples were then stored at 90 °C in an electric oven. The apparent viscosities of the XG and XG-L solutions were measured every day, and the apparent viscosities of the XG-HC solution and XG-LC solutions were measured every 4 d. A viscometer (DV2TLV; Brookfield, Middleboro, MA, USA) was used to measure viscosity after each sample was stirred with a no. 62 rotor at 60 rpm for 1 min. Except for day 0, all experimental temperatures were above 80 °C. Once the apparent viscosity of a sample dropped below 10 mPa·s, it was considered to have completely lost its viscosity, and no more viscosity measurements were taken for that sample. Storage time was used as an index for thermal stability evaluation. All apparent viscosity measurements were performed in triplicate.

### 2.7. Structural Characterization of Xanthan Gum Samples

The FTIR spectra of different xanthan gum samples were determined using the method described by Ngwabebhoh et al. (2021) [27], with some modifications. Briefly, the xanthan gum solutions prepared in Section 2.6 were freeze-dried and crushed into a powder. The chemical structures of XG, XG-L, XG-HC, and XG-LC were elucidated based on the FTIR spectra (Tensor II; Bruker, Karlsruhe, Germany) through the wavenumber range of 4000–400 cm^−1^. The scanning resolution was 4 cm^−1^, and 32 scans were performed. Furthermore, SEM images of the above four xanthan gum samples were obtained using a JSM-7610F Plus field emission scanning electron microscope (JEOL, Akishima-shi, Tokyo, Japan) at an acceleration voltage of 5 kV and a magnification of 10,000×. To improve the conductivity of the samples, they were sprayed with gold before capturing the SEM images [28].

## 3. Results

### 3.1. Basic Properties of Natural and Pyruvate-Free Xanthan Gum Samples

**Figure 1:**
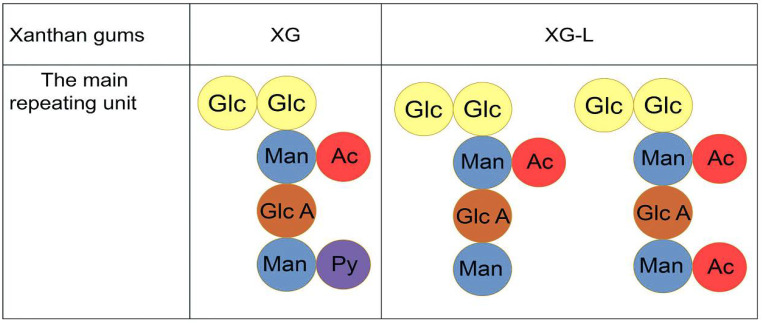
Main repeating units of natural xanthan gum (XG) and pyruvate-free xanthan gum (XG-L). Glc, glucose; Man, mannose; Glc A, glucuronic acid; Ac, acetyl group; Py, pyruvyl group.

The main structures of XG and XG-L are shown in Figure 1. The theoretical maximum content of acetyl groups in xanthan gum was 12.52% if both acetylation sites are full of acetyl groups [25]. However, the acetyl content of XG-L was 7.04% (Table 2), suggesting that XG-L may have two main structures (Figure 1).

### 3.2. Thermal Stabilities of Different Xanthan Gum Solutions

**Figure 2:**
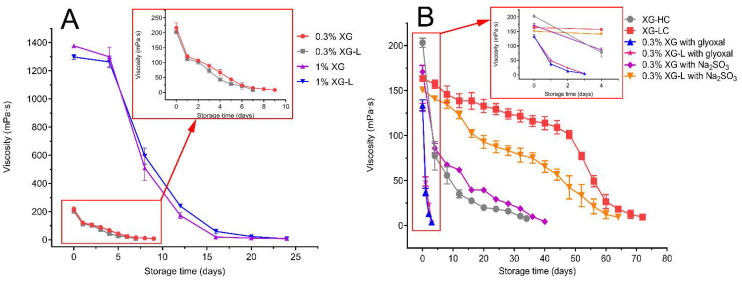
(**A**) Thermal stability of XG and XG-L at different concentrations; (**B**) Thermal stability of XG-HC, XG-LC, XG, and XG-L with Na_2_SO_3_ or glyoxal. XG, natural xanthan gum; XG-L, pyruvate-free xanthan gum; XG-HC, natural xanthan gum with a deoxidizer and glyoxal added; XG-LC pyruvate-free xanthan gum with a deoxidizer and glyoxal added.

As seen in Figure 2A, the thermal stability of XG-L was not improved compared with XG, at two concentrations (1% and 0.3%). No significant difference in the storage time required to lose viscosity was found between XG and XG-L samples.

The storage time at which XG-HC lost viscosity was 34 days, which was significantly longer than the time taken for XG and XG-L to lose viscosity (Figure 2B). However, the thermal stability of XG-LC was significantly improved compared with both XG and XG-L (Figure 2B). The apparent viscosity of the 0.3% (*m*/*v*) XG-LC solution remained above 100 mPa·s for 48 days of storage at 90 °C, which was much longer the amount of time that the XG, XG-L, and XG-HC solutions remained viscous, and more than twice the amount of time that the XG-HC solution remained viscous. When XG (day 9) and XG-HC completely lost their viscosity, the viscosity of XG-LC had only decreased by 11% and 26%, respectively. On day 52, the apparent viscosity of XG-LC decreased below 100 mPa·s, and the loss of viscosity accelerated slightly.

Moreover, after adding Na_2_SO_3_ to XG, its thermal stability significantly increased (Figure 2B), but it was not significantly different from the thermal stability of XG-HC. In contrast, the thermal stability of XG-LC was significantly greater than that of XG-L mixed only with Na_2_SO_3_. Throughout the storage time, the apparent viscosity was always higher for XG-LC than XG-L mixed only with Na_2_SO_3_. However, when glyoxal was added to XG and XG-L, the thermal stability of the two samples decreased sharply, and viscosity was completely lost within 4 days (Figure 2B).

### 3.3. Effects of Pyruvyl Groups and Stabilizers on the Structure of Xanthan Gum

**Figure 3:**
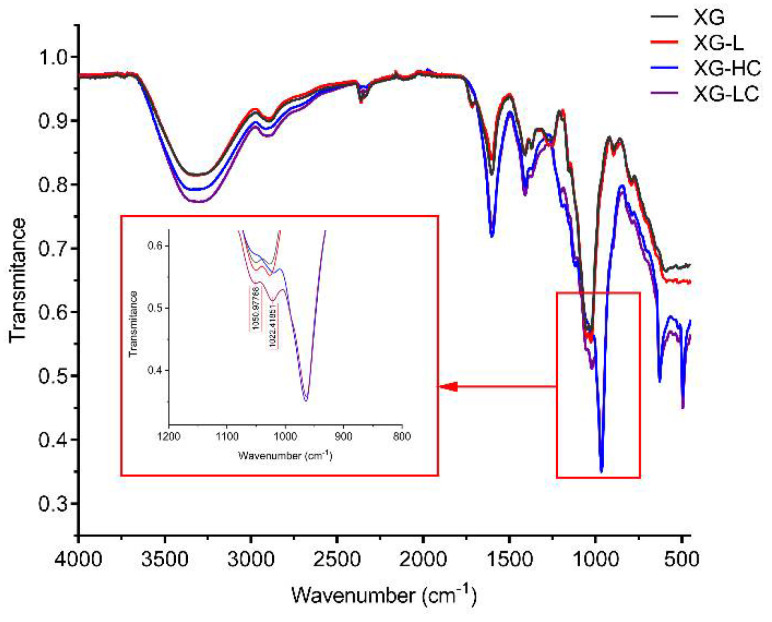
Fourier-transform infrared spectra of XG, XG-L, XG-HC, and XG-LC. XG, natural xanthan gum; XG-L, pyruvate-free xanthan gum; XG-HC, natural xanthan gum with a deoxidizer and glyoxal added; XG-LC pyruvate-free xanthan gum with a deoxidizer and glyoxal added.

**Figure 4:**
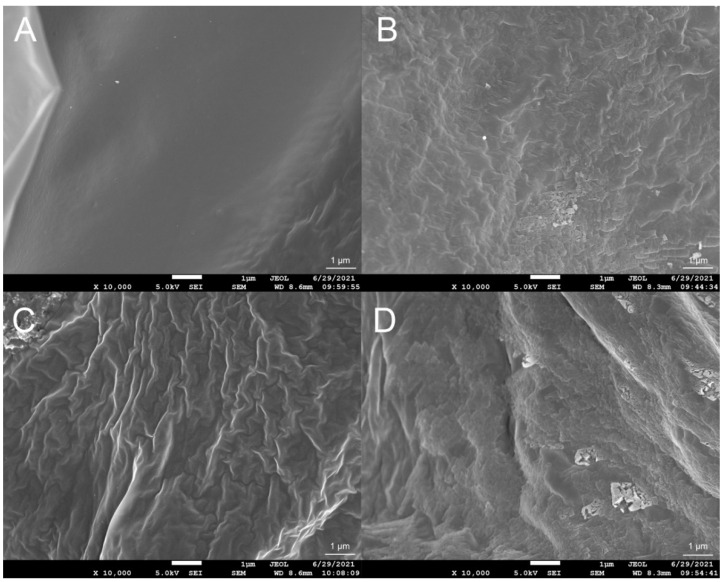
Scanning electron microscopic images of (**A**) XG, (**B**) XG-HC, (**C**) XG-L, and (**D**) XG-LC. XG, natural xanthan gum; XG-HC, natural xanthan gum with a deoxidizer and glyoxal added; XG-L, pyruvate-free xanthan gum; XG-LC pyruvate-free xanthan gum with a deoxidizer and glyoxal added.

The FTIR spectra of the xanthan gum samples are shown in Figure 3. All samples showed a broad intense absorption peak at 3200–3500 cm^−1^, due to the stretching vibration of O-H. The band at 2800–3000 cm^−1^ was due to the C-H stretching vibration. The peaks at 1600 cm^−1^ and 1410 cm^−1^ indicated the asymmetric and symmetric stretching vibrations of the C-O bonds of the carboxyl group, respectively. Three peaks at 960 cm^−1^, 630 cm^−1^, and 490 cm^−1^ only existed in XG-LC and XG-HC spectra and, thus, may be attributable to Na_2_SO_3_. Two small peaks were also observed at 1050 cm^−1^ and 1022 cm^−1^. These may be attributable to C-O or C-O-C stretching vibrations from the glycosidic bonds of xanthan gum [29]. It is worth noting that these two peaks were higher for the XG-LC sample. This minor difference may be crucial to the positive effect of glyoxal on the thermal stability of XG-L, as discussed in the Discussion section. SEM images indicated that the surface structures of the four xanthan gum samples were significantly different (Figure 4). The XG sample exhibited a flat surface at 10,000× magnification, while the surface of XG-L appeared wrinkled (Figure 4C). However, a scale-like structure was observed on the surfaces of both XG-HC and XG-LC (Figure 4B,D), with XG-LC showing a distinct scale-like structure.

## 4. Discussion

Xanthan gum is an important microbial polysaccharide with various uses. Maintaining the viscosity of xanthan gum at high temperatures is of great importance for its applications in the petroleum industry. In this study, we aimed to obtain a xanthan gum product with enhanced thermal stability through genetic engineering technology combined with stabilizer treatment and to assess the mechanism responsible for the thermal stability enhancement. Therefore, we constructed the XC-Δ*gumL* strain of *X. campestris* and added Na_2_SO_3_ and glyoxal to the xanthan gum preparations to obtain different types of xanthan gum products. The thermal stability of the xanthan gum samples was then measured. Furthermore, the effects of pyruvyl deletion and stabilizer addition on the structure of each xanthan gum sample were also evaluated.

Xanthan gum has an ordered spiral structure at the conformational transition temperature, but when the temperature increases, the structure becomes disordered [30]. It is widely accepted that the conformational transition temperature may be increased by depyruvylation. This change in conformational transition temperature is considered one of the indicators used to evaluate the thermal stability of xanthan gum [30]. However, the key indicator for evaluating the thermal stability of xanthan gum is the maintenance of viscosity under long-term storage at high temperatures [13]. Our experimental results showed that although the structure of the xanthan gum samples changed, there was no significant change in their viscosity characteristics under continuous storage at high temperatures. XG-L showed no change in thermal stability at high or low concentrations. We speculate that two main internal factors may have led to this result. First, in the structure of xanthan gum, acetyl is conducive to the stability of ordered conformation, pyruvyl destroys the stability of ordered conformation [31,32]. Moreover, pyruvate promotes intermolecular interactions between different molecules of xanthan gum [31]. The content of acetyl increases (Table 2), and a single xanthan gum molecule will form a more ordered and stable helical conformation when pyruvate is absent. This may reduce the number of intermolecular interactions, affecting the overall thermal stability. Second, other characteristics of the xanthan gum product, such as chain length, may also affect its thermal stability [30]. These characteristics may also be affected when pyruvate transferase is deactivated in the producer strain, eventually causing a change in thermal stability.

Therefore, in response to this situation, we explored strategies to increase the viscosity-retention time of xanthan gum at sustained high temperatures. Previous studies have identified two main reasons for the loss of viscosity of xanthan gum solutions at high temperatures. First, oxidation reactions, which are the main cause of xanthan gum degradation, readily occur at high temperatures and lead to a loss of viscosity [14]. Second, polymer hydrolysis, which occurs under acidic or basic conditions, is also accelerated at high temperatures [12]. Therefore, we added Na_2_SO_3_ and glyoxal to xanthan gum samples to investigate their protective effects on the maintenance of viscosity at high temperatures. Na_2_SO_3_ is a typical deoxidizer that is widely used to inhibit oxidation reactions [33]. Although glyoxal is neither a deoxidizer nor a buffer salt, it is a widely used cross-linking agent, as it has two active aldehyde groups [34,35]. Theoretically, glyoxal can cross-link xanthan gum molecules in solution. Moreover, in previous studies, glyoxal has been reported to improve the thermal stability of polysaccharide solutions [15]. 

Our results indicated that glyoxal alone had no significant effect on the thermal stability of xanthan gum, whereas the addition of Na_2_SO_3_ without glyoxal was an effective strategy for thermal stability enhancement (Figure 2B). However, when Na_2_SO_3_ and glyoxal were added simultaneously, the thermal stability of the natural xanthan gum samples did not change significantly compared to the addition of Na_2_SO_3_ alone. This finding was inconsistent with the results of previous studies, and it prompted us to explore the mechanism. We found that the five polysaccharides (scleroglucan, Wellan gum, diutan, rhamZan, and succinoglycan) reported to show improved thermal stability after the addition of Na_2_SO_3_ and glyoxal are non-ionic or weak anionic polysaccharides that are rich in hydroxyl groups [36,37,38,39,40]. In contrast, xanthan gum is a typical anionic polysaccharide in its natural form [41]. This may be why the simultaneous addition of Na_2_SO_3_ and glyoxal had little effect on its thermal stability. However, when pyruvate is eliminated, the anionic content of xanthan gum is expected to decrease significantly, and, thus, it would become a weak anionic polysaccharide. Therefore, we added Na_2_SO_3_ and glyoxal simultaneously to the XG-L sample, and this significantly improved its thermal stability. 

Another question arising from these results is why this strategy was effective for the XG-L sample but ineffective for the XG sample. As we mentioned above, the anionic content of XG-L is significantly lower than the anionic content of XG. Therefore, the xanthan gum molecules in XG-L were closer to weakly acidic glyoxal molecules, which is crucial for glyoxal to function as a cross-linking bridge to link the xanthan gum molecules. Comparatively, the more potent repulsive force of the negative charges in the XG solution hindered this effect. The FTIR spectra of different samples support this conclusion. Compared with the other samples, the C-O and C-O-C stretching vibrations (1050 cm^−1^ and 1022 cm^−1^, respectively) of XG-LC were enhanced compared with the other samples (Figure 3), suggesting that acetal or hemiacetal formed between xanthan gum and glyoxal [28,42,43]. Glyoxal was used as a cross-linking agent and could react with the hydroxyl groups in xanthan gum to form acetal or hemiacetal. There are multiple hydroxyl groups in either the main chain or the side chain of xanthan gum, and all of them could act as the sites on which the cross-linked structure formed. Furthermore, the removal of the pyruvate group in the side chain of xanthan gum resulted in an additional hydroxyl site in the side chain (the acetyl substitution degree of this site was low, as shown in Table 2), which improved the possibility of cross-linking between xanthan gum and glyoxal. The cross-linking between xanthan gum and glyoxal could be similar to that between cellulose and glyoxal, in which the intermolecular cross-linking exists as the main form [44]. Furthermore, SEM images showed distinctly different surface morphologies of the different samples. The wrinkles that appeared on the surface of XG-L may also have been caused by the close association of the side chains with the backbone. The formation of a scaly structure was clearly related to the presence of glyoxal and Na_2_SO_3_.

It was reported that Na2SO3 alone also had a positive impact on the thermal stability of xanthan gum [14]. Therefore, we measured the thermal stability of xanthan gum with Na_2_SO_3_ or glyoxal alone to investigate their respective effects. However, the use of glyoxal alone harmed thermal stability. We believe that this was caused by the oxalic acid generated by the oxidation of glyoxal. When Na_2_SO_3_ is not added, glyoxal tends to oxidize to oxalic acid in the solution, making the xanthan gum solution acidic. Most polysaccharides are easily hydrolyzed under acidic conditions [14,45], so we found that the thermal stability of xanthan gum was significantly reduced. However, when Na_2_SO_3_ was added to the solution, it protected xanthan gum from oxidation and had a significant antioxidant effect on glyoxal. In this case, glyoxal could play the role of cross-linking agent and improve the thermal stability of xanthan gum.

To verify this hypothesis, we used the Fehling test to determine the aldehyde groups in the solution. We found that after being stored at 90 °C for 24 h, the pH of the solution with glyoxal alone dropped from 6.4 to 3.4, and there was almost no red precipitate during the Fehling reagent titration process. Meanwhile, the pH value of the solution with Na_2_SO_3_ was maintained at about 7.3, and a large amount of red precipitate was produced. This indicated that the addition of Na_2_SO_3_ makes glyoxal in a stable state and can play the role of cross-linking agent. However, there is also another possibility that glyoxal reacted with the Na_2_SO_3_ to form glyoxal bisulfite [46,47], which might affect the thermal stability of xanthan gum solution in a different manner. Further research is needed to detailly investigate the effect of glyoxal bisulfite on the thermal stability of xanthan gum.

## 5. Conclusions

In summary, the purpose of this study was to establish a long-term viscosity maintenance strategy for xanthan gum stored continuously at high temperatures and to investigate the mechanism responsible for the enhanced thermal stability of xanthan gum. We constructed the mutant *X. campestris* strain, XC-Δ*gumL*, through genetic engineering to produce the XG-L sample. We then added a deoxidizer, Na_2_SO_3_, and glyoxal during the production of xanthan gum to prepare XG-LC and XG-HC samples. Modifying the structure of xanthan gum so that it lacked pyruvate and then adding glyoxal and Na_2_SO_3_ during the production process significantly improved the thermal stability of xanthan gum. The elimination of pyruvate from xanthan gum may have reduced its negative charge and brought glyoxal molecules closer to the pyruvate-free xanthan gum molecules, thus allowing glyoxal to cross-link the xanthan gum molecules. The deoxidizer, Na_2_SO_3_, protected both xanthan gum and glyoxal from being oxidized during this process. The strategy we developed to improve the thermal stability of xanthan gum may contribute to its applications in processes that require long periods of time at high temperatures, such as the tertiary oil recovery process used in the petroleum industry. Moreover, these results lay a foundation for future investigations of the underlying mechanisms.

## Figures and Tables

**Table 1:** Primers used in this study.

Primers	Sequence
*gumL*-F1	CGCGGATCCATGGCCAACGCTTTACTGCAGAA
*gumL*-R1	AGGCCGTGCGCTGGAATCTTG
*gumL*-F2	GATTCCAGCGCACGGCCT
*gumL*-R2	CCCAAGCTTTCACCACAAATCGTAAGGGAACGCAGC

**Table 2:** Basic characteristics of natural xanthan gum and xanthan gum from genetically engineered Xanthomonas campestris strains.

Samples	Pyruvyl (wt.%) ^b^	Acetyl (wt.%) ^b^	MW (Da)	Yield (g/100 g)	Producing Strain
XG ^a^	3.86 ± 0.08	6.35 ± 0.06	1.83 × 10^7^	3.05 ± 0.08	XC
XG-L ^a^	0.06 ± 0	7.04 ± 0.11	1.67 × 10^7^	2.90 ± 0.03	XC-Δ*gumL*

^a^ XG, natural xanthan gum; XG-L, pyruvate-free xanthan gum; XC, X. campestris NRRL B-1459; wt., weight percentage. ^b^ Contents of pyruvyl and acetyl were measured in triplicate, and data were presented as the mean ± standard deviations.

## Data Availability

The data presented in this study are available on request from the corresponding author.
